# Serotype Changes and Drug Resistance in Invasive Pneumococcal Diseases in Adults after Vaccinations in Children, Japan, 2010–2013

**DOI:** 10.3201/eid2111.142029

**Published:** 2015-11

**Authors:** Kimiko Ubukata, Naoko Chiba, Shigeo Hanada, Miyuki Morozumi, Takeaki Wajima, Michi Shouji, Satoshi Iwata, Invasive Pneumococcal Diseases Surveillance Study Group

**Affiliations:** Keio University School of Medicine, Tokyo, Japan (K. Ubukata, N. Chiba, M. Morozumi, S. Iwata);; Toranomon Hospital, Tokyo (S. Hanada);; Tokyo University of Pharmacy and Life Sciences, Tokyo (T. Wajima);; National Cancer Center Hospital, Tokyo (M. Shouji)

**Keywords:** invasive pneumococcal diseases, adult, serotypes, vaccines, PPSV23, PCV7, PCV13, MLST, drug resistance, antimicrobial resistance, bacteria, respiratory infections, streptococci, Japan

## Abstract

After 7-valent pneumococcal conjugate vaccine (PCV) for children was introduced in Japan in November 2010, we examined changes in *Streptococcus pneumoniae* serotypes and in genetic antimicrobial drug resistance of isolates from adults with invasive pneumococcal diseases. During April 2010–March 2013, a total of 715 isolates were collected from adults with invasive pneumococcal diseases. Seven-valent PCV serotypes in adults decreased from 43.3% to 23.8%, most noticeably for serotype 6B. Concomitantly, 23-valent pneumococcal polysaccharide vaccine (PPSV23) serotypes decreased from 82.2% to 72.2%; non-PPSV23 serotypes increased from 13.8% to 25.1%. Parallel with serotype changes, genotypic penicillin-resistant *S. pneumoniae* decreased from 32.4% to 21.1%, and 6 non-PPSV23 serotypes emerged (6D, 15A, 15C, 16F, 23A, and 35B). Respective vaccine coverage rates for 13-valent PCV and PPSV23 differed by disease: 73.9% and 84.3% for patients with pneumonia, 56.4% and 69.2% for patients with bacteremia and sepsis, and 45.7% and 69.3% for patients with meningitis.

## Abstract 2

Pneumococcal conjugate vaccination of children is associated with penicillin-resistant *Streptococcus pneumoniae* in adults.

*Streptococcus pneumoniae* can cause invasive pneumococcal diseases (IPDs), which can be treated with generally effective antimicrobial drugs. In adults, well-known risk factors for IPDs include underlying conditions such as splenectomy, functional asplenia, immunodeficiency, and HIV infection. Risk is also increased with age >65 years, diabetes, renal dialysis, and chronic hepatic dysfunction (*1**,**2*).

In 2010, the US Advisory Committee on Immunization Practices (ACIP) issued recommendations that all persons >65 years of age should be vaccinated with 23-valent pneumococcal polysaccharide vaccine (PPSV23) to prevent pneumococcal diseases (*3*). In 2012, the ACIP also recommended that 13-valent pneumococcal conjugate vaccine (PCV13), which the US Food and Drug Administration approved in 2011, be given to adults >19 years of age if they have immunocompromising conditions (*4*). Recently, the ACIP recommended routine administration of both PCV13 and PPSV23 to all adults >65 years of age (*5*).

In the United States, 7-valent pneumococcal conjugate vaccine (PCV7) has been used to vaccinate children since 2000, resulting in individual and herd immunity and a decline in pneumococcal infection in children (*6**–**9*). After PCV7 was introduced, serotype 19A pneumococcal strains with penicillin resistance increased (*8**–**10*); the change in 2012 to PCV13 covers serotype 19A (*11*).

Presently, pneumococcal conjugate vaccines (PCVs) are incorporated into pediatric vaccination schedules in >120 countries, and PCV13 has been approved for adults in >100 countries. Pneumococcal infections in adults have decreased as an indirect effect of widespread use of PCVs for children (*2**,**6**,**8**,**9**,**12*). However, in countries where PCV7 or PCV13 was introduced, overall coverage of serotypes by the vaccine gradually decreased because of pneumococcal serotype replacement from vaccine type to nonvaccine type. In particular, increases of nonvaccine serotypes, such as 6C, 15A, 23A, and 35B, have been reported in the United States (*13**,**14*) and other countries (*15**–**18*).

In Japan, PCV7 vaccination was introduced for children <5 years of age in November 2010 by the Provisional Fund for the Urgent Promotion of Vaccination. PCV7 was incorporated into routine vaccination schedules in April 2013 and replaced by PCV13 in November 2013. PCV7 rapidly decreased IPD infections in children. However, pneumococcal infections caused by non-PCV7 serotypes (19A, 15A, 15B, 15C, and 24) increased in children during 2012 (*19*).

Meanwhile, Japan became the first “super-aging” society in the world in 2013, when 25.1% of the nation’s population was >65 years of age (Statistics Bureau, Ministry of Internal Affairs and Communications, http://www.stat.go.jp/english/data/handbook/c0117.htm). Lifestyle-related and other diseases that affect response to infection have increased in older adults, and pneumonia has again become the third leading cause of death in Japan. Nevertheless, the voluntary PPSV23 immunization rate among older persons long remained <18%. In November 2014, the Japanese Ministry of Health, Labour and Welfare began promoting routine vaccination with PPSV23 for adults >65 years of age. 

Because of these developments, we sought to clarify changes in serotypes and in genetic antimicrobial resistance in isolates from adults with IPDs after PCV7 for children was introduced in Japan. We describe capsular serotypes, drug-resistance genotypes, and multilocus sequence typing in isolates from adults with IPDs, and we examine the relationships of these factors to specific IPDs.

## Methods

### Patients and Pneumococcal Strains

Our study included adult patients (>19 years of age) with IPD. Pneumococcal isolates from sterile clinical samples such as blood, cerebrospinal fluid, pleural effusion, and joint fluid were collected from clinical laboratories at 341 hospitals participating in this IPD surveillance study. Each hospital had a microbiology laboratory, and the hospitals were distributed nearly uniformly throughout Japan. The hospitals participated in the surveillance project after written permission was obtained from laboratory or hospital directors.

From April 2010 through March 2013, a total of 715 pneumococcal isolates were collected for the study: 275 during April 2010–March 2011, the first surveillance period; 213 during April 2011–March 2012, the second period; and 227 during April 2012–March 2013, the third period. This large-scale IPD surveillance for adults was performed in conjunction with IPD surveillance for children (*19*). During the first surveillance period, the rate of voluntary PCV7 vaccination for children was ≈10%. The second period occurred simultaneously with the Urgent Promotion of Vaccination incentive for children, and the immunization rate was ≈50%–60%. The third period occurred just before the transition from PCV7 to PCV13 for children, and the PCV7 immunization rate was ≈80%–90%. However, the rate of voluntary vaccination with PPSV23 in adults remained <18% throughout the surveillance periods.

The collected pneumococcal isolates were sent promptly from each clinical laboratory to our laboratory (Molecular Epidemiology for Infectious Agents, Kitasato Institute for Life Sciences, Kitasato University, Tokyo, Japan), accompanied by survey forms completed by attending physicians but not identifying patients in compliance with ethical guidelines for epidemiology in Japan. The surveys collected information on patient’s age at illness onset, sex, clinical manifestations, blood test results at hospitalization, and outcome. Clinical manifestations and diagnoses were verified in all patients, according to diagnostic criteria and guidelines for sepsis and related conditions (*20*).

### Serotypes and Antimicrobial Drug–Resistant Genotypes

Serotypes were determined by the capsular quellung reaction by using antiserum purchased from the Statens Serum Institute (Copenhagen, Denmark). By using real-time PCR methods described previously (*21*), alterations were identified in 3 penicillin-binding protein (PBP) genes mediating β-lactam resistance in *S. pneumoniae*: *pbp1a* (encoding enzyme PBP1A), *pbp2x* (encoding PBP2X), and *pbp2b* (encoding PBP2B). Amino acid substitutions within or near each PBP’s conserved amino acid motifs, such as serine-threonine (Thr)–methionine-lysine (STMK), were detected by using real-time PCR. The *mef* (A) and *erm* (B) genes mediating macrolide resistance were also identified by real-time PCR (*21*). Quinolone resistance was analyzed by sequencing the quinolone-resistance–determining region in the genes *gyrA*, *gyrB*, *parC*, and *parE* in strains with MICs of levofloxacin of >4 μg/mL (*22*).

Gene analysis identified genotypes (g) on the basis of their responses to β-lactam antimicrobial drugs: penicillin-susceptible *S. pneumoniae* (gPSSP) possessing 3 normal *pbp* genes; penicillin-intermediate *S. pneumoniae* (gPISP), also classified as gPISP (*pbp2x*), gPISP (*pbp2b*), gPISP (*pbp1a+pbp2x*), or gPISP (*pbp2x+pbp2b*); and penicillin-resistant *S. pneumoniae* (gPRSP), which possessed all 3 abnormal *pbp* genes (*19**,**21*). Macrolide-resistant genotypes were also represented variously: macrolide-susceptible *S. pneumoniae* that possessed no resistance genes; macrolide resistance mediated by the *mef*(A) gene, MLR-*mef*(A); macrolide resistance mediated by the *erm*(B) gene, MLR-*erm*(B); and macrolide resistance involving both genes, MLR-*mef*(A)+*erm*(B).

Identification of capsular type by the quellung reaction and resistance genotyping by real-time PCR was performed within 1 day of sample collection for each strain. Results were reported immediately to the medical staff at the referring hospital.

### Antimicrobial Susceptibility Testing

MICs of 7 intravenous antimicrobial agents (penicillin, ampicillin, cefotaxime, ceftriaxone, meropenem, panipenem, and vancomycin) were determined for all pneumococcal isolates by agar-dilution methods (*23*). We obtained each agent from the respective manufacturer.

### Multilocus Sequence Typing

Multilocus sequence typing (MLST; http://www.MLST.net) was performed for nonvaccine serotype strains, except for PPSV23 serotypes, according to previously described methods with minor modifications (*24*). Primers used were based on sequences listed by the US Centers for Disease Control and Prevention (http://www.cdc.gov/ncidod/biotech/strep/alt-MLST-primers.htm). Analyses by using MLST and eBURST (Department of Infectious Disease Epidemiology, Imperial College of London, UK) were performed, as described by the MLST website (http://spneumoniae.mlst.net).

### Statistical Analysis

We assessed differences in serotypes by age groups, clinical symptoms and signs, resistance types, and surveillance periods. To determine whether differences were statistically significant, we performed χ^2^ tests or the Fisher exact test by using Ekuseru-Toukei 2012 software for statistics (Social Survey Research Information, Tokyo, Japan). 

## Results

### Yearly Changes in Pneumococcal Serotypes

During each annual surveillance period (April–March) during 2010–2013, pneumococcal serotypes from IPD isolates varied (Table 1). All strains were isolated from sterile samples, such as blood, cerebrospinal fluid, pleural effusion, or joint fluid.

**Table 1:** Pneumococcal serotypes in isolates from adult patients with invasive pneumococcal diseases, Japan, April 2010–March 2013*

Vaccine serotype	2010–2011, no. (%), N = 275	2011–2012, no. (%), N = 213	2012–2013, no. (%), N = 227	p value†
PCV7				
4	14 (5.1)	17 (8.0)	7 (3.1)	0.075
6B	41 (14.9)	24 (11.3)	12 (5.3)	0.003
9V	7 (2.5)	5 (2.3)	1 (0.4)	0.168
14	21 (7.6)	16 (7.5)	17 (7.5)	0.998
18C	1 (0.4)	1 (0.5)	1 (0.4)	ND
19F	14 (5.1)	8 (3.8)	7 (3.1)	0.511
23F	21 (7.6)	13 (6.1)	9 (4.0)	0.232
Total	119 (43.3)	84 (39.4)	54 (23.8)	<0.001
PCV13				
1	1 (0.4)	1 (0.5)	0	ND
3	45 (16.4)	27 (12.7)	42 (18.5)	0.226
5	0	0	1 (0.4)	ND
6A	11 (4.0)	4 (1.9)	6 (2.6)	0.367
7F	9 (3.3)	2 (0.9)	3 (1.3)	0.128
19A	18 (6.5)	11 (5.2)	17 (7.5)	0.593
Total	203 (73.8)	129 (60.6)	123 (54.2)	<0.001
PPSV23				
10A	10 (3.6)	8 (3.8)	11 (4.8)	0.754
11A	3 (1.1)	8 (3.8)	5 (2.2)	0.142
15B	3 (1.1)	4 (1.9)	5 (2.2)	0.605
22F	10 (3.6)	19 (8.9)	18 (7.9)	0.040
Other‡	8 (2.9)	1 (0.5)	8 (3.5)	0.082
Total	226 (82.2)	165 (77.5)	164 (72.2)	0.036
Nonvaccine serotype				
6C	13 (4.7)	12 (5.6)	17 (7.5)	0.407
15A	6 (2.2)	10 (4.7)	8 (3.5)	0.310
15C	0	4 (1.9)	4 (1.8)	ND
23A	2 (0.7)	8 (3.8)	8 (3.5)	0.053
35B	7 (2.5)	6 (2.8)	9 (4.0)	0.626
37	3 (1.1)	0	1 (0.4)	ND
38	3 (1.1)	2 (0.9)	3 (1.3)	0.928
Other§	3 (1.1)	2 (0.9)	5 (2.2)	0.454
Total	37 (13.5)	44 (20.7)	55 (24.2)	0.007
Nontypeable¶	1 (0.4)	0	2 (0.9)	

*Years run from April 1 to March 31 of the following year. ND, not determined because of a low number of strains. †p values characterize comparisons of 2010–2011, 2011–2012, and 2012–2013. ‡Serotypes 12F, 20, and 33F. §Serotypes 6D, 7C, 16F, 18B, 24F, and 34. ¶These serotypes were excluded from the statistical analysis because they were unclassifiable.

During all surveillance periods, the estimated voluntary inoculation rate for PPSV23 in adults was <18% in Japan. In February 2010, the vaccination incentive for PCV7 in children began on a voluntary basis. In November 2010, the Special Fund for the Urgent Promotion of Vaccination was initiated, and PCV7 vaccination of children <5 years of age became an official priority throughout Japan. Estimates of the PCV7 immunization rate in children was <10% in 2010, 50%–60% in 2011, and 80%–90% in 2012 (*19*).

PPSV23 coverage of serotypes found in IPD isolates decreased significantly each year, from 82.2% in 2010 to 77.5% in 2011 to 72.2% in 2012 (p = 0.036). Respective coverages with PCV7 and PCV13 also decreased significantly, from 43.3% and 73.8% in 2010 to 39.4% and 60.6% in 2011 to 23.8% and 54.2% in 2012, respectively (p<0.001). Although serotype 22F increased significantly in IPD isolates, yearly decreases of serotype 6B contributed markedly to decreases in PPSV23 coverage. Nonvaccine serotypes (i.e., non-PPSV23) increased significantly during the surveillance periods from 13.5% in 2010 to 20.7% in 2011 to 24.2% in 2012 (p = 0.007).

Introduction of PCV7 in children indirectly affected adults with IPD, shown by changes in each serotype during 2010–2012 (Technical Appendix Figure 1). Decreases occurred in PCV7 serotypes and serotype 6A, the latter of which exhibits cross-immunity with 6B, but almost all other serotypes except those in PCV13 increased, especially serotypes 22F, 6C, 23A, 15C, 15A, and 35B.

### Yearly Changes in Genotypes Resistant to Penicillin, Macrolides, and Quinolones

Genotypes resistant to penicillin, macrolides, and quinolones varied during the surveillance periods (Table 2). Among genotypes resistant to penicillin, 40% were gPISP (*pbp2x*), 26% were gPRSP, 10.2% were gPISP (*pbp1a*+*pbp2x*), and 5.9% were gPISP (*pbp2x*+*pbp2b*). Only 16.1% were gPSSP strains without any abnormal *pbp* genes. Other genotypes were gPISP (*pbp2b*; 1.3%) and gPISP (*pbp1a+2b*; 0.6%). In contrast to a gradual increase in gPISP (*pbp2x*+*pbp2b*; p = 0.025), gPRSP decreased significantly over the surveillance periods (p = 0.008). In addition, 89.7% of all pneumococcal strains possessed *mef*(A) or *erm*(B) genes that mediated macrolide resistance: 23.6% carried the *mef*(A) gene mediating intermediate macrolide resistance; 56.9% carried the *erm*(B) gene mediating high macrolide resistance; and 9.1% possessed both genes. Macrolide-susceptible strains accounted for only 10.3%. Furthermore, only 0.7% of strains were highly resistant to levofloxacin (MIC >16 μg/mL) and possessed mutations in both *gyrA* and *parC* genes. Intermediate-resistance strains (MIC 4 μg/mL) that possessed the *parC* mutation accounted for 1.3% of strains.

**Table 2:** Isolates positive for invasive pneumococcal diseases and resistant to penicillin, macrolide, and quinolone, by genotype, Japan, April 2010–March 2013*

Genotype	Total, no. (%), N = 715	2010–2011, no. (%), n = 275	2011–2012, no. (%), n = 213	2012–2013, no. (%), n = 227	p value†
Penicillin resistance					
gPSSP	115 (16.1)	42 (15.3)	40 (18.8)	33 (14.5)	0.431
gPISP (*pbp2x*)	286 (40.0)	96 (34.9)	89 (41.8)	101 (44.5)	0.076
gPISP (*pbp2b*)	9 (1.3)	5 (1.8)	0	4 (1.8)	ND
gPISP (*pbp1a+2x*)	73 (10.2)	30 (10.9)	23 (10.8)	20 (8.8)	0.701
gPISP (*pbp1a+2b*)	4 (0.6)	3 (1.1)	1 (0.5)	0	ND
gPISP (*pbp2x+2b*)	42 (5.9)	10 (3.6)	11 (5.2)	21 (9.3)	0.025
gPRSP (*pbp1a+2x+2b*)	186 (26.0)	89 (32.4)	49 (23.0)	48 (21.1)	0.008
Macrolide resistance					
Resistance gene (-)	74 (10.3)	34 (12.4)	16 (7.5)	24 (10.6)	0.216
*mef*(A) gene	169 (23.6)	63 (22.9)	56 (26.3)	50 (22.0)	0.538
*erm*(B) gene	407 (56.9)	152 (55.3)	123 (57.7)	132 (58.1)	0.777
*mef*(A) and *erm*(B)	65 (9.1)	26 (9.5)	18 (8.5)	21 (9.3)	0.925
Quinolone resistance‡					
* gyrA+parC*	5 (0.7)	2 (0.7)	3 (1.4)	0	ND
* parC*	9 (1.3)	5 (1.8)	1 (0.5)	3 (1.3)	ND

*Years run from April 1–March 31 of the following year. ND, not determined because of a low number of strains. †p values characterize comparison of the 3 surveillance periods. ‡Quinolone resistance, Ser_81_Phe substitution in GyrA and Ser_79_Tyr in ParC, respectively.

### Yearly Changes in Resistance Genotypes and Serotypes

Yearly changes in genotypes and serotypes affecting β-lactam resistance also occurred during the 3 periods (Figure). Decreases closely related to a reduction of serotypes 6B, 19F, and 23F, but not of serotype 14, were found for a number of gPRSP. Emergence of gPRSP was evident among 6 non-PPSV23 serotypes (6D, 15A, 15C, 16F, 23A, and 35B), although such resistance strains were few. Almost all non-PPSV23 serotype strains showed macrolide resistance (Technical Appendix Figure 2), which did not change significantly from year to year (Table 2).

**Figure:**
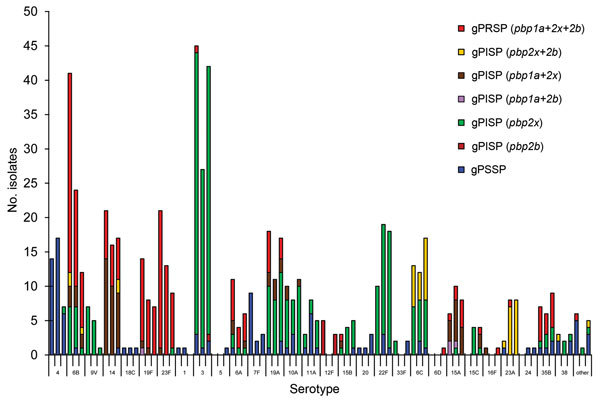
Yearly changes in number of serotypes and in penicillin resistance in genotypes found in isolates from adults with invasive pneumococcal diseases, Japan, April 2010–March 2013. Serotypes are shown for each of the 3 yearly surveillance periods: April 2010–March 2011, April 2011–March 2012, and April 2012–March 2013. Short tic marks on horizontal axis represent yearly number of isolates for specific serotypes; longer tic marks represent the 3-year surveillance period for each serotype. gPSSP, genotypic penicillin-susceptible *Streptococcus pneumoniae*; gPISP, genotypic penicillin-intermediate resistant *S. pneumoniae*; gPRSP, genotypic penicillin-resistant *S. pneumoniae*. Parentheses enclose abnormal *pbp* genes that mediate penicillin resistance.

### Invasive Pneumococcal Diseases and Patient Age

To examine relationships between IPDs and patient age at illness onset we classified IPDs were classified into 4 categories (Table 3): pneumonia (n = 375), including cases of empyema (n = 17) and pleuritis (n = 16); bacteremia and sepsis with no obvious focus (n = 172), including sepsis (n = 51), severe sepsis (n = 57), and septic shock (n = 24); meningitis (n = 127); and others (n = 41), including cellulitis (n = 7), arthritis (n = 6), spondylitis (n = 4), and peritonitis (n = 3). Pneumonia (52.4%) was the most common category of IPDs, followed by bacteremia and sepsis (24.1%) and meningitis (17.8%). Mean age of patients with meningitis was 65 years, younger than those with pneumonia or bacteremia and sepsis (mean age 74 and 70 years, respectively). Meningitis occurred significantly more often among patients <64 years of age, in contrast to pneumonia, which occurred most often in persons >75 years of age (p<0.001).

**Table 3:** Isolates positive for invasive pneumococcal diseases in adults, by age group, Japan, April 2010–March 2013

Disease	No. (%)						Mean age, y (±SD)	p value
Total	19–49	50–64	65–74	75–84	>85
Pneumonia*	375 (52.4)	33 (39.8)	66 (42.0)	103 (49.8)	105 (60.7)	68 (71.6)	74 (14.9)	<0.001
Bacteremia and sepsis†	172 (24.1)	20 (24.1)	42 (26.8)	55 (26.6)	34 (19.7)	21 (22.1)	70 (15.0)	0.497
Meningitis	127 (17.8)	24 (28.9)	38 (24.2)	37 (17.9)	22 (12.7)	6 (6.3)	65 (14.4)	<0.001
Other‡	41 (5.7)	6 (7.2)	11 (7.0)	12 (5.8)	12 (6.9)	0	67 (14.5)	0.133
Total	715	83	157	207	173	95	70 (14.9)	

*Includes empyema (n = 17) and pleuritis (n = 16). †Bacteremia (n = 39), sepsis (n = 51), severe sepsis (n = 58), and septic shock (n = 24). ‡Cellulitis (n = 7), arthritis (n = 6), spondylitis (n = 4), peritonitis (n = 3), and other conditions.

Data for deaths and survival were available for 656 (91.7%) of the 715 patients. Proportions of deaths occurring within 30 days of hospitalization increased significantly with age: 6.0% at 19–49 years, 17.8% at 50–64 years, 17.9% at 65–74 years, 25.4% at 75–84 years, and 26.3% at >85 years (p = 0.002). Overall rate of death occurring within 30 days after hospitalization was 21.3%; overall rate for serious sequelae was 10%.

### Serotype Distribution by Disease

A comparison of proportions of serotypes included in PCV7, PCV13, PPSV23, and non-PPSV23 by disease category (i.e., pneumonia, bacteremia and sepsis, meningitis, and others) showed that frequency of serotypes differed considerably by type of IPD (Table 4). Prevalence of serotypes 3, 14, and 19A was higher in isolates of patients with pneumonia than in those from patients with other illnesses (p value range <0.001–0.076). In contrast, serotypes 10A, 15A, 23A, and 23F were significantly more prevalent in isolates of patients with meningitis than in those from patients with other illnesses (p value range <0.001–0.004).

**Table 4:** Pneumococcal serotypes by type of invasive pneumococcal diseases in adults, Japan, April 2010–March 2013*

Serotype	Pneumonia, no. (%), n = 375	Bacteremia and sepsis, no. (%), n = 172	Meningitis, no. (%), n = 127	Others, no. %, n = 41	Total, no. (%), n = 715	p value†
PCV7 serotypes						
4	25 (6.7)	8 (4.7)	5 (3.9)	0	38 (5.3)	0.403
6B	42 (11.2)	20 (11.6)	11 (8.7)	4 (9.8)	77 (10.8)	0.667
9V	10 (2.7)	3 (1.7)	0	0	13 (1.8)	0.161
14	36 (9.6)	9 (5.2)	6 (4.7)	3 (7.3)	54 (7.6)	0.076
18C	1 (0.3)	1 (0.6)	1 (0.8)	0	3 (0.4)	ND
19F	14 (3.7)	9 (5.2)	4 (3.1)	2 (4.9)	29 (4.1)	0.617
23F	16 (4.3)	7 (4.1)	15 (11.8)	5 (12.2)	43 (6.0)	0.004
Total	144 (38.4)	57 (32.6)	42 (32.3)	14 (34.1)	257 (35.5)	0.325
Additional PCV13 serotypes						
3	84 (22.4)	17 (9.9)	7 (5.5)	6 (14.6)	114 (15.9)	<0.001
6A	7 (1.9)	9 (5.2)	4 (3.1)	1 (2.4)	21 (2.9)	0.101
7F	10 (2.7)	2 (1.2)	2 (1.6)	0	14 (2.0)	0.463
19A	32 (8.5)	9 (5.2)	3 (2.4)	2 (4.9)	46 (6.4)	0.035
Other‡	0	3 (1.7)	0	0	3 (0.4)	ND
Total	277 (73.9)	97 (56.4)	58 (45.7)	23 (56.1)	455 (63.6)	<0.001
Additional PPSV23 serotypes						
10A	6 (1.6)	8 (4.7)	11 (8.7)	4 (9.8)	29 (4.1)	0.001
11A	10 (2.7)	3 (1.7)	3 (2.4)	0	16 (2.2)	0.798
15B	4 (1.1)	4 (2.3)	2 (1.6)	2 (4.9)	12 (1.7)	0.533
22F	20 (5.3)	12 (7.0)	12 (9.4)	3 (7.3)	47 (6.6)	0.269
Other§	6 (1.6)	4 (2.3)	6 (4.7)	1 (2.4)	17 (2.4)	0.139
Total¶	316 (84.3)	119 (69.2)	88 (69.3)	32 (78.0)	555 (77.6)	<0.001
Nonvaccine serotypes						
6C	21 (5.6)	10 (5.8)	7 (5.5)	4 (9.8)	42 (5.9)	0.994
15A	5 (1.3)	9 (5.2)	9 (7.1)	1 (2.4)	24 (3.4)	0.003
15C	1 (0.3)	4 (2.3)	3 (2.4)	0	8 (1.1)	ND
23A	2 (0.5)	7 (4.1)	8 (6.3)	1 (2.4)	18 (2.5)	<0.001
35B	7 (1.9)	9 (5.2)	4 (3.1)	2 (4.9)	22 (3.1)	0.101
38	6 (1.6)	1 (0.6)	1 (0.8)	0	8 (1.1)	ND
Other#	7 (1.9)	4 (2.3)	3 (2.4)	0	14 (2.0)	0.918
Total	49 (13.1)	44 (25.6)	35 (27.6)	8 (19.5)	136 (19.0)	<0.001
Nontypeable**	3 (0.8)	0	0	0	3 (0.4)	

*ND, not determined because of low number of strains. PCV7, 7-valent pneumococcal conjugate vaccine; PCV13, 13-valent pneumococcal conjugate vaccine; PPSV23, 23-valent pneumococcal polysaccharide vaccine. †Significant difference obtained by comparing pneumonia, sepsis, and meningitis. ‡Serotypes 1 and 5. §Serotypes 12F, 20, and 33F. ¶Serotype 6A is not included in PPSV23. #Serotypes 6D, 7C, 16F, 18B, 24F, 34, and 37. **Serotypes that were excluded from the statistical analysis because they were unclassifiable.

Coverages for vaccines among all isolates collected and examined during the study duration were 35.5% for PCV7, 63.6% for PCV13, and 77.6% for PPSV23. Respective coverages of PCV13 and PPSV23 differed significantly for each IPD group: 73.9% and 84.3% for pneumonia, 56.4% and 69.2% for bacteremia and sepsis, 45.7% and 69.3% for meningitis, and 56.1% and 78.0% for others (p<0.001 for all). The most prevalent serotype was serotype 3 (15.9%), followed by serotypes 6B (10.8%), 14 (7.6%), 22F (6.6%), 19A (6.4%), and 23F (6.0%). Death rates did not differ significantly by pneumococcal serotype.

### Antimicrobial Drug Susceptibility by Genotype

The Technical Appendix Table 1 shows susceptibilities (50% MIC, 90% MIC, and MIC range) of 6 parenteral β-lactam agents for *S. pneumoniae* strains (n = 710). PBP gene alterations affect MICs of penicillin, ampicillin, cefotaxime, and meropenem (Technical Appendix Figure 3). Most strains in this study were gPISP with *pbp2x* gene alterations that reduced susceptibility to cephalosporin agents rather than to penicillin. MICs of cefotaxime and ceftriaxone were affected more by *pbp2x* alterations than by *pbp2b* alterations. In contrast, MICs of penicillin, ampicillin, and meropenem were affected more by *pbp2b* alterations than by *pbp2x* alterations. Strains showing MICs >4μg/mL to cefotaxime had *pbp2a* gene alteration and also had *pbp1a*, *pbp2x*, and *pbp2b* gene alterations (data not shown).

### MLST in Non-PCV13 Serotype Strains

MLST results for 92 strains (non-PCV13 serotypes) collected during 2012 and results for 2 gPRSPs (serotypes 15B collected during 2010 and 23A collected during 2011) indicate that sequence types (STs), 8 of which were new, and clonal complexes (CCs) varied (Table 5). We identified 3 new STs of gPRSPs possessing macrolide-resistant genes: ST6138 (CC81) in serotype 15C, ST8351 (CC3117) in serotype 16F, and ST9619 (CC156) in serotype 23A. Also, we identified gPRSPs of ST282 (CC81) in serotype 6D, reported from South Korea in 2008 and derived from Vietnam serotype 23F in 1996; ST63 (CC63) in serotype 15A, derived from Sweden^15A^-25 in 1992; ST83 (CC81) in serotype 15B, reported from South Korea in 2007 and derived from Taiwan serotype 23F in 1997; and ST558 (CC558) in serotype 35B, reported from the United States in 1999 (Table 5). We confirmed that all gPRSPs examined in our study had abnormal PBPs by DNA sequencing: substitutions of Thr371Serine or Thr371Alanine (Ala) within conserved amino acid motif STMK in PBP1A; Thr338Ala within STMK in PBP2X; and Thr445Ala adjacent to the serineserine–asparagine amino acid motif in PBP2B, respectively.

**Table 5:** Serotypes, resistance genotypes, and multilocus sequence type for non-PCV13 serotype isolates from adults with invasive pneumococcal diseases, Japan, April 2010–March 2013*

Serotype (total no. isolates)	No. isolates, N = 92	Penicillin resistance genotype	Macrolide resistance gene	Clonal complex	ST	Earliest report of same ST†	
						Year	Country (city)
6C (17)	1	gPISP(*pbp2x+2b*)	*erm*(B)	315	386	1996	Portugal (Lisbon)
	4	gPISP(*pbp2x*)	*erm*(B)	2924	2924	2003	Japan (Hyogo)
	2	gPISP(*pbp2x*)	*erm*(B)	2924	6183	2008	Japan (Kanagawa)
	1	gPISP(*pbp2x*)	*erm*(B)	2924	**9336‡**	2012	Japan (Fukuoka)
	1	gPSSP	Non	473	473	1994	England (Oxford)
	3	gPISP(*pbp2x+2b*)	*erm*(B)	156	5241	2008	Japan (Kumamoto)
	2	gPISP(*pbp2x+2b*)	*mef*(A)	5832	**5025‡**	2012	Japan (Kanagawa)
	2	gPISP(*pbp2x+2b*)	*mef*(A) or *erm*(B)	5832	5832	2009	Japan (Chiba)
	1	gPISP(*pbp2x+2b*)	*mef*(A)+*erm*(B)	5832	5832	2009	Japan (Chiba)
6D (1)	1	gPRSP	*mef*(A)	81	282	1996; 2008	Vietnam [23F]; South Korea [6D]
10A (11)	1	gPISP(*pbp2x*)	Non	156	1263	1998	USA (Arizona/New Mexico)
	1	gPISP(*pbp2x*)	Non	156	**3395‡**	2012	Japan (Toyama)
	8	gPISP(*pbp2x*)	*erm*(B)	113	5236	2007	Japan (Kanagawa)
	1	gPISP(*pbp1a+2x*)	*erm*(B)	113	**3078‡**	2012	Japan (Yamaguchi)
11A (5)	1	gPSSP	*mef*(A)	99	99	2007	South Korea (Seoul)
	4	gPISP(*pbp2x*)	*mef*(A)+*erm*(B)	99	99	2007	South Korea (Seoul)
15A (8)	4	gPISP*(pbp1a+2x*)	*erm*(B)	63	63	1992	[Sweden^15A^-25]
	4	gPRSP	*erm*(B)	63	63	1992	[Sweden^15A^-25]
15B (6)	1	gPSSP	*mef*(A)	199	199	1987	[Netherlands^15B^-37]
	3	gPISP(*pbp2x*)	*erm*(B)	199	199	1987	[Netherlands^15B^-37]
	1	gPISP(*pbp2x*)	*erm*(B)	199	**5609‡**	2012	Japan (Tokyo)
	1	gPRSP	*erm*(B)	81	83	1997 2007	Taiwan [23F] South Korea [15]
15C (4)	1	gPISP*(pbp2x*)	*erm*(B)	199	199	1987	[Netherlands^15B^-37]
	2	gPISP(*pbp1a+2x*)	*erm*(B)	199	199	1987	[Netherlands^15B^-37]
	1	gPRSP	*erm*(B)	81	**6138‡**	2013	Japan (Tokyo)
16F (1)	1	gPRSP	*mef*(A)	3117	**8351‡**	2011	Japan (Mie)
20 (3)	3	gPSSP	*erm*(B)	4745	4745	2005	China (Wuhan, Hubei)
22F (18)	1	gPSSP	Non	433	433	1997	Poland (center)
	4	gPISP(*pbp2x*)	Non	433	433	1997	Poland (center)
	10	gPISP(*pbp2x*)	*mef*(A) or *erm*(B)	433	433	1997	Poland (center)
	1	gPISP(*pbp2x*)	*mef*(A)	113	5236	2007	Japan (Kanagawa)
	2	gPISP(*pbp2x*)	*erm*(B)	113	7158	2007	Japan (Aichi)
23A (9)	2	gPISP(*pbp2x+2b*)	*erm*(B)	156	338	1995	[Colombia^23F^-26]
	4	gPISP(*pbp2x+2b*)	*erm*(B)	156	5242	2009	Japan (Kumamoto)
	2	gPISP(*pbp2x+2b*)	*erm*(B)	156	5246	2008	Japan (Niigata)
	1	gPRSP	*erm*(B)	156	**9619‡**	2011	Japan (Toyama)
35B (9)	2	gPSSP	*erm*(B)	1816	2755	2004	China (Shanghai)
	2	gPISP(*pbp2x*)	*mef*(A) or *erm*(B)	1816	2755	2004	China (Shanghai)
	1	gPRSP	Non	558	558	1999	USA (New York) [35B]
	4	gPRSP	*mef*(A) or *erm*(B)	558	558	1999	USA (New York) [35B]

*PCV13, 13-valent pneumococcal conjugate vaccine; ST, sequence type. Brackets in final column represent clones reported in the Pneumococcal Molecular Epidemiology Network Clone Collection (http://web1.sph.emory.edu/PMEN/index.html).  †Earliest year and first location are shown for the applicable ST registered in the Multilocus Sequence Typing website (http://spneumoniae.mlst.net).  **‡**Identified as a new ST in this study.

## Discussion

Wide use of PCV7 or PCV13 in young children has contributed to a decline in IPD (*6**–**9*) and other pneumococcal diseases, including pneumonia (*25**,**26*) and otitis media (*27**,**28*). The decline resulted from both direct vaccine effect and herd immunity.

In the United States, introduction of PCV7 led to decreased rates of IPD, resulting from vaccine-related strains in children and in unvaccinated adults because of indirect effects of pediatric use (*6**,**29**,**30*). After increased incidence of serotype 19A among causative pathogens in children, multivalent PCV13 replaced PCV7 in 2010 (*11*). PCV13 now has been introduced in >120 countries, and various pneumococcal diseases have decreased as a result. Unfortunately, serotype replacement by non-PCV13 serotypes such as 6C, 15B, 22F, 23A, and 35B has also ensued (*13**,**14**,**16**–**18**,**31*).

In Japan, incorporation of PCV7 and *Haemophilus influenzae* type b conjugate vaccine into routine vaccination schedules for children occurred later than in other countries and was spurred by the Provisional Special Fund for the Urgent Promotion of Vaccination, initiated in November 2010. The vaccination rate for children improved rapidly from <10% in 2010 to 50%–60% in 2011 and 80%–90% in 2012. Routine PCV7 for children was implemented officially in April 2013 and was replaced with PCV13 in November 2013.

Although PPSV23 was approved in 1988, few (<18% in 2012) adults voluntarily received PPSV23 vaccination. Since October 2014, vaccination with PPSV23 in adults >65 years of age has been supported by public government funding. However, at this time, vaccination rates are unclear because these older persons can receive 1 PPSV23 vaccination over a 5-year period.

In our large-scale surveillance, we aimed to clarify molecular epidemiology for pneumococci and β-hemolytic streptococci and to collect background data on patients with IPD throughout Japan during April 2010–March 2013. Pediatric IPD caused by PCV7 serotypes and other PCV7-related 6A serotypes decreased significantly soon after vaccine implementation (*19*), but the non-PCV7 serotypes 19A, 15A, 15B, 15C, and 24 increased each year. These results reflect simultaneous replacement of PCV7 serotypes with non-PCV7 serotypes among pneumococci that colonize the nasopharynx of children (K. Ubukata et al., unpub. data). Consequently, frequency of transmission of PCV7 serotypes from children to adults would be expected to decrease.

In this study, we found epidemiologic changes among pneumococcal strains isolated from adult patients with IPD; vaccine-type strains decreased significantly. However, the prominent decrease in serotype 6B detected by our surveillance differed from findings in the United States, where serotype 14 decreased most prominently after PCV7 introduction (*14*). IPD cases in Japan resulting from serotype 14 decreased significantly for adults during our ongoing surveillance in 2014. Given Japan’s high population density, effects of herd immunity were likely present in children and adults. However, non-PCV7 types, especially serotype 22F, increased.

A decrease in penicillin-nonsusceptible *S. pneumoniae* strains has been associated with serotype changes occurring after vaccine introduction (*29**,**30**,**32*). In our previous genetic studies, the gPRSP with 3 PBP alterations encoded by the *pbp1a*, *pbp2x*, and *pbp2b* genes also decreased significantly (*19*). However, our current study found increases in the gPISP with *pbp2x* gene alterations that reduce susceptibilities to cephalosporin agents (e.g., cefotaxime and ceftriaxone), rather than reducing susceptibility to penicillin. This trend likely reflects preference among medical practices in Japan for cephalosporin and macrolide agents.

We also identified some gPRSPs not previously reported as new STs. Of 7 STs identified in gPRSP and non-PCV7 serotypes, 4 STs had been posted in the pneumococcal database of the MLST website: ST558 (CC558) of serotype 35B, identified in the United States (*33*); ST63 (CC63) of serotype 15A derived from Sweden^15A^-25; ST282 (CC81) of serotype 6D in South Korea (*34**,**35*); and ST83 (CC81) of serotype15B, originating in Taiwan serotype 23F. The remaining 3 STs identified in gPRSP, serotypes 15C, 16F, and 23A, appear to be novel STs arising in Japan. ST6138 of serotype 15C and ST9619 of serotype 23A were estimated to be extended from ST83 and ST338 (Pneumococcal Molecular Epidemiology Network clone Colombia^23F^-26), respectively (*36*). 

Recent pneumococcal genomic analysis (*37**,**38*) indicated occurrence of capsular switching by genetic recombination among strains of different serotypes. Recombination of regions consisting of the capsular *cps* gene locus and 2 adjacent *pbp1a* and *pbp2x* genes results in capsular switching and penicillin resistance. A pneumococcal strain arising from such an event will show high penicillin resistance from mutations in *pbp* genes because of selection pressure from antimicrobial drugs. MLST and genotypic results of gPRSPs identified in non-PCV13 strains in this study support occurrence of such genetic events.

In relation to serotypes and IPDs in adults, PPSV23 coverage has been reported to differ by disease (*39*). Our results show respective coverages of PCV13 and PPSV23 to be 73.9% and 84.3% for patients with pneumonia and 45.7% and 69.3% for patients with meningitis. This finding supports recommendations by the ACIP (*5*) for routine vaccinations with both PCV13 and PPSV23 in all adults >65 years of age, especially those with serious underlying diseases. For pneumococci with a large number of capsular types, development of a novel vaccine against components other than the capsule is expected (*40*). Overuse of antimicrobial agents should be avoided worldwide to avoid selection of novel resistant strains capable of undermining vaccine efficacy.

**Technical Appendix.** Isolate susceptibility to 6 antimicrobial agents and increases and decreases in serotype by annual surveillance period, changes in serotypes and macrolide resistance, and distribution of susceptibilities to 4 antimicrobial agents for invasive pneumococcal diseases in adults, Japan, April 2010–March 2013.
