# Supplementary material for: Serotype Changes and Drug Resistance in Invasive Pneumococcal Diseases in Adults after Vaccinations in Children, Japan, 2010–2013
Source: Emerg Infect Dis. 2015 Nov;21(11):1956–65. doi: 10.3201/eid2111.142029 (PMC4622236; doi:10.3201/eid2111.142029)
Supplement: Supplementary file 1 — Technical Appendix. Isolate susceptibility to 6 antimicrobial agents and increases and decreases in serotype by annual surveillance period, changes in serotypes and macrolide resistance, and distribution of susceptibilities to 4 antimicrobial agents for invasive pneumococcal diseases in adults, Japan, April 2010–March 2013. [file 14-2029-Techapp-s1.pdf]

Article DOI: http://dx.doi.org/10.3201/eid2111.142029

Serotype Changes and Drug Resistance in Invasive Pneumococcal Diseases in Adults after Vaccinations in Children, Japan, 2010–2013

Technical Appendix

Technical Appendix Table. Susceptibility to 6 parenteral β-lactam antimicrobial agents by genotype class in isolates from adults with invasive pneumococcal diseases, Japan, April 2010–March 2013.

	Concentration (µg/mL)		
Antimicrobial agent and genotype class*	MIC_{50}	MIC_{90}	MIC range
Penicillin			
gPSSP (n = 115)	0.016	0.031	0.004–0.031
gPISP (*pbp2x*) (n = 285)	0.063	0.063	0.016–0.125
gPISP (*pbp1a+2x*) (n = 73)	0.125	0.25	0.063–1
gPISP (*pbp2x+2b*) (n = 41)	0.125	0.5	0.031–0.5
gPRSP (*pbp1a+2x+2b*) (n = 184)		2	0.25–4
Ampicillin			
gPSSP	0.031	0.031	0.004–0.031
gPISP (*pbp2x*)	0.063	0.125	0.008–0.125
gPISP (*pbp1a+2x*)	0.125	0.5	0.031–1
gPISP (*pbp2x+2b*)	0.25	0.5	0.063–1
gPRSP (*pbp1a+2x+2b*)	1	2	0.125–4
Cefotaxime			
gPSSP	0.016	0.031	0.008–0.25
gPISP (*pbp2x*)	0.25	0.5	0.008–0.5
gPISP (*pbp1a+2x*)	1	1	0.125–4
gPISP (*pbp2x+2b*)	0.25	0.5	0.031–0.5
gPRSP (*pbp1a+2x+2b*)	1	2	0.125–8
Ceftriaxone			
gPSSP	0.016	0.063	0.008–0.125
gPISP (*pbp2x*)	0.25	0.25	0.016–0.5
gPISP (*pbp1a+2x*)	0.5	1	0.25–1
gPISP (*pbp2x+2b*)	0.25	0.25	0.063–0.5
gPRSP (*pbp1a+2x+2b*)	0.5	1	0.25–2
Meropenem			
gPSSP	0.016	0.031	0.008–0.063
gPISP (*pbp2x*)	0.016	0.063	0.008–0.125
gPISP (*pbp1a+2x*)	0.063	0.125	0.016–0.25
gPISP (*pbp2x+2b*)	0.063	0.5	0.016–0.5
gPRSP (*pbp1a+2x+2b*)	0.5	1	0.063–4
Panipenem			
gPSSP	0.004	0.004	0.002–0.008
gPISP (*pbp2x*)	0.004	0.008	0.002–0.016
gPISP (*pbp1a+2x*)	0.016	0.016	0.004–0.063
gPISP (*pbp2x+2b*)	0.016	0.031	0.004–0.031
gPRSP (*pbp1a+2x+2b*)	0.063	0.125	0.008–0.25

*Results of gPISP (*pbp2b*) (n = 9) and gPISP(*pbp1a+2b*) (n = 3) were omitted because of a low number of strains.

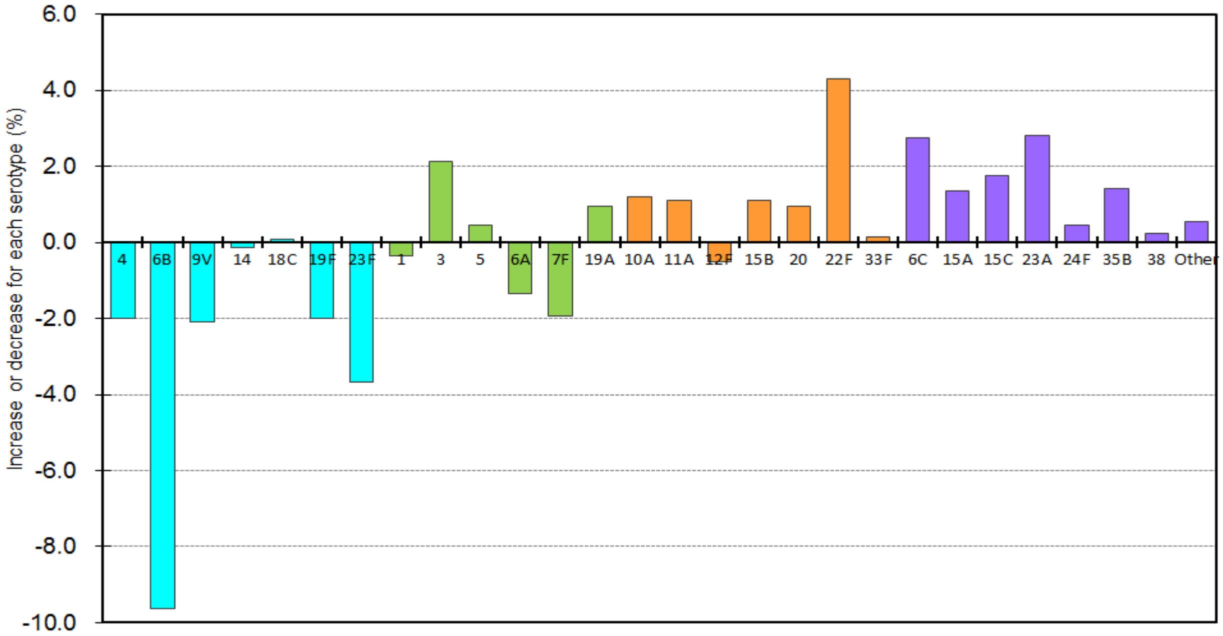

Technical Appendix Figure 1. Comparison of proportional increases and decreases in serotypes in isolates from adults with invasive pneumococcal diseases, Japan, from first surveillance period (April 2010–March 2011) to third surveillance period (April 2012–March 2013). Blue indicates serotypes included in PCV7; green indicates serotypes included in PCV13; orange indicates serotypes included in PPSV23; and purple indicates serotypes not included in PPSV23.

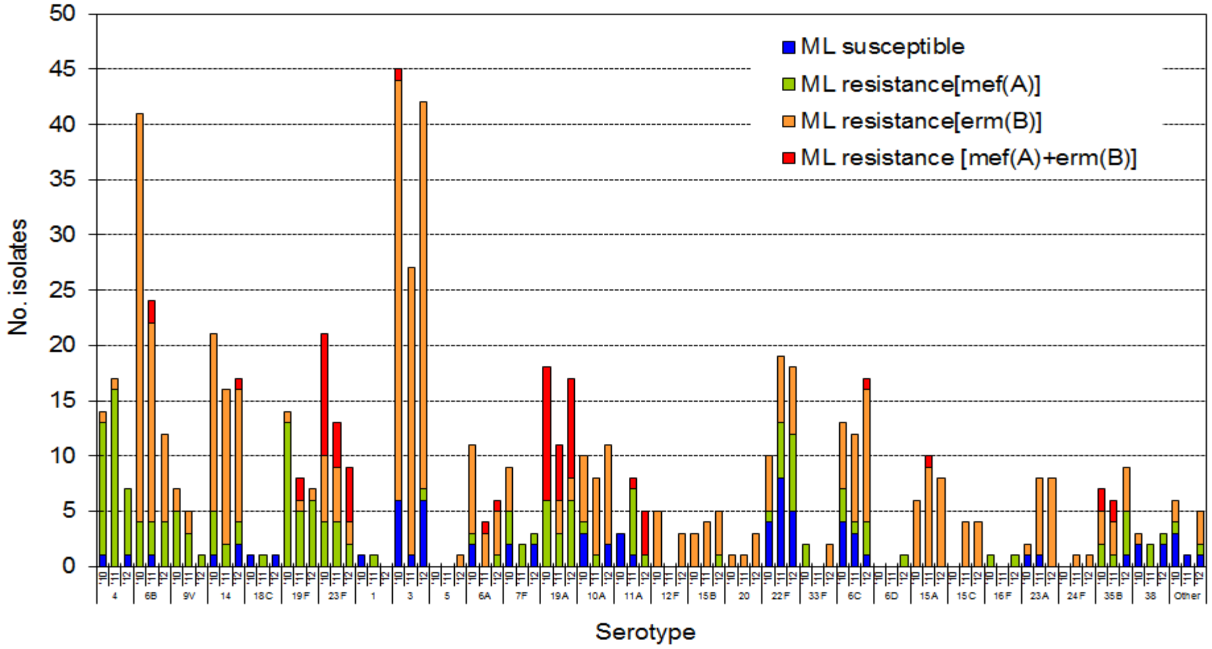

Technical Appendix Figure 2. Changes in number of serotypes and in macrolide (ML) resistance by genotype in isolates from adults with invasive pneumococcal diseases for 3 yearly surveillance periods, Japan, April 2010–March 2013. ML susceptible indicates not possessing any ML-resistance gene; ML resistance [*mef*(A)] indicates possessing *mef*(A) gene mediating ML-intermediate resistance to 14-membered MLs and azalide; ML resistance [*erm*(B)] indicates possessing *erm*(B) gene mediating ML-high resistance; and ML resistance [*mef*(A)+*erm*(B)] indicates possessing both resistance genes.

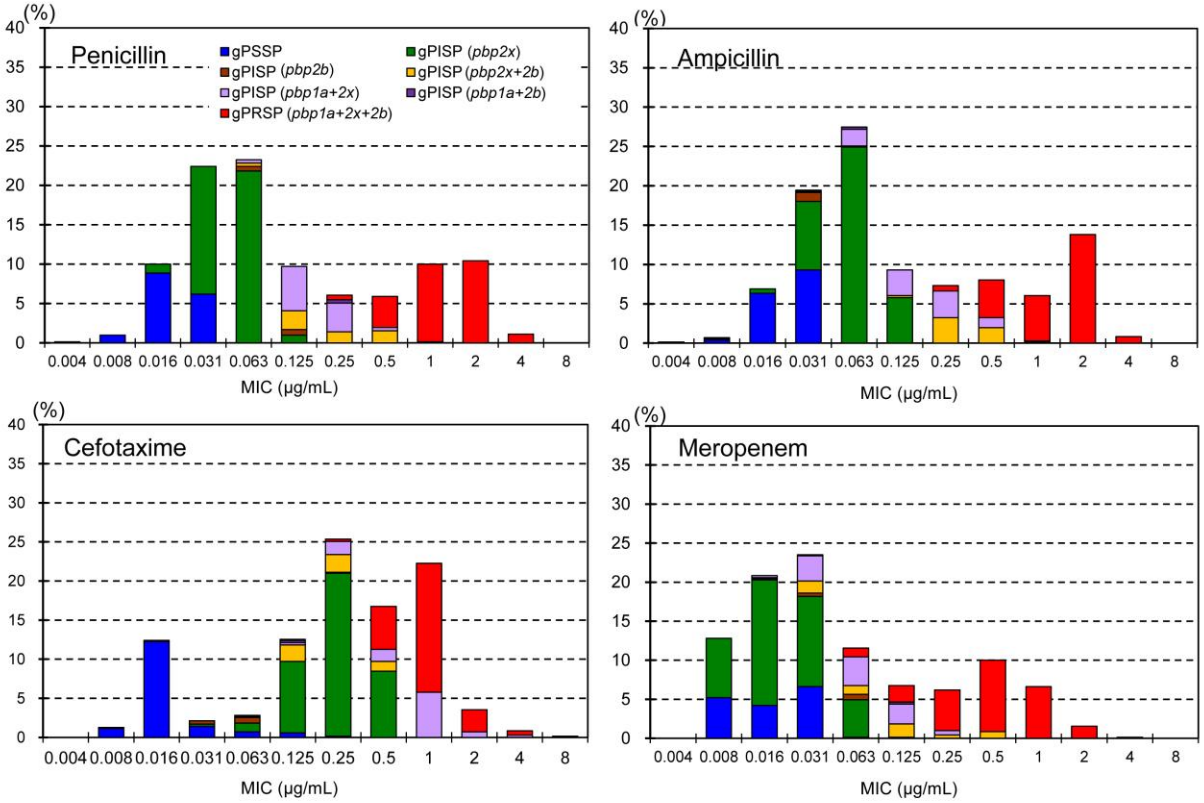

Technical Appendix Figure 3. Distribution of susceptibilities to 4 parenteral β-lactam antimicrobial agents (penicillin, ampicillin, cefotaxime, and meropenem) by genotype in isolates from adults with invasive pneumococcal diseases, Japan, April 2010–March 2013. gPSSP indicates genotypic penicillin-susceptible *Streptococcus pneumoniae*; gPISP indicates genotypic penicillin-intermediate–resistant *S. pneumoniae*; and gPRSP indicates genotypic penicillin-resistant *S. pneumoniae*. Parentheses enclose abnormal *pbp* genes mediating penicillin resistance.
